# Enhanced Electrochemical Performance Promoted by Tin in Silica Anode Materials for Stable and High-Capacity Lithium-Ion Batteries

**DOI:** 10.3390/ma14051071

**Published:** 2021-02-25

**Authors:** Xuli Ding, Daowei Liang, Hongda Zhao

**Affiliations:** School of Science, Jiangsu University of Science and Technology, 666 Changhui Road, Zhenjiang 212100, China; Leong1664988857@163.com (D.L.); zhd1787278660@163.com (H.Z.)

**Keywords:** porous carbon, silica, tin, anode materials, lithium-ion batteries

## Abstract

Although the silicon oxide (SiO_2_) as an anode material shows potential and promise for lithium-ion batteries (LIBs), owing to its high capacity, low cost, abundance, and safety, severe capacity decay and sluggish charge transfer during the discharge–charge process has caused a serious challenge for available applications. Herein, a novel 3D porous silicon oxide@Pourous Carbon@Tin (SiO_2_@Pc@Sn) composite anode material was firstly designed and synthesized by freeze-drying and thermal-melting self-assembly, in which SiO_2_ microparticles were encapsulated in the porous carbon as well as Sn nanoballs being uniformly dispersed in the SiO_2_@Pc-like sesame seeds, effectively constructing a robust and conductive 3D porous Jujube cake-like architecture that is beneficial for fast ion transfer and high structural stability. Such a SiO_2_@Pc@Sn micro-nano hierarchical structure as a LIBs anode exhibits a large reversible specific capacity ~520 mAh·g^−1^, initial coulombic efficiency (ICE) ~52%, outstanding rate capability, and excellent cycling stability over 100 cycles. Furthermore, the phase evolution and underlying electrochemical mechanism during the charge–discharge process were further uncovered by cyclic voltammetry (CV) investigation.

## 1. Introduction

Lithium-ion batteries (LIBs) have been regarded as one of the critical energy storage technologies that can be widely used in portable electronics and grid-scale energy storage due to their high energy density and cycle longevity to make a fossil fuel-free environment possible [1,2,3,4,5]. With the advent of electric vehicles (EV) in recent years, the traditional commercialized LIBs are obviously insufficient to meet the requirement owning to their limited capacities. Therefore, it is highly desired for LIBs with higher energy and power densities as well as lower cost to be developed [6,7,8].

According to the working principle of LIBs, the electrode materials play a critical role in the further improvement of the battery performance [9,10,11,12,13,14,15,16]. High-capacity and low-cost materials have triggered vast interest in the past few years [15,16,17,18,19,20,21], which can bring great promise for next-generation LIBs with a higher price–performance ratio. Silica (SiO_2_) has recently captured great attention as a promising candidate anode materials for LIBs because of its suitable working potential (~0.25 V vs. Li/Li^+^), proper theoretical specific capacity (~1960 mAh·g^−1^), lesser volume variation (~100%), and expanded cycling lifespan compared to silicon and other alloys [22,23,24,25,26,27,28,29,30,31,32,33,34,35,36,37,38,39]. In addition, SiO_2_ is one of the most abundant materials on earth, and its environmentally friendly and low-cost nature further turns it into an alternative electrode material [25,26,27,28,29,30]. However, the development of SiO_2_-based anode materials so far has been impeded due to its poor electrical conductivity and sluggish charge transfer kinetics. To overcome these limitations, extensive research efforts have been dedicated to the development of SiO_2_-based composite materials and structures, such as carbon-coated SiO_2_ particles [32,35,36], SiO_2_/Cu polyacrylonitrile-C composite [33], Sn(SnO_2_)–SiO_2_/graphene nanocomposites [37], Bi_2_S_3_@SiO_2_ core-shell microwires [38], Ni/SiO_2_ hierarchical hollow spheres [39], and so on [34,40]. Even though significant progress has been achieved, the commercialization of SiO_2_-based anodes is still restricted by the low electrochemical activity. On the other hand, metallic tin (Sn) has a good electrical conductivity (8.7 × 10^6^ S·m^−1^) and low melting point (~232 °C). When used as an electrode material, it shows a high theoretical capacity of 994 mAh·g^−1^ with the formation of Li_4.4_Sn and fitting working potential (~0.5 V). However, the huge volume changes (~260%) it suffers during Li alloying/dealloying can always lead to rapid fading of capacity and subsequently poor cyclability [41,42,43,44,45,46,47].

Taking advantages of both SiO_2_ and Sn, herein, a feasible tactics was developed to construct porous silicon oxide@Pourous Carbon@Tin (SiO_2_@Pc@Sn) composites with tunable SiO_2_ to Sn molar ratios to synergistically storage Li in both porous SiO_2_ and Sn. The SiO_2_@Pc@Sn composite was fabricated using a simple and scalable freezing-drying and low-temperature thermal-melting combined method. The obtained composites possessed several advantageous features: Firstly, the porous structure in the composites largely shortened the transport path for Li ions and provided the buffering space for volume change during the charging/discharging process; secondly, porous C (Pc) and SiO_2_ provided a rigid skeleton with long cycle stability; thirdly, the presence of Sn and Pc could improve the electrical conductivity of the SiO_2_-based electrode. The synergetic effect of porous SiO_2_, Pc, and Sn nano-ball empowered the fabricated SiO_2_@Pc@Sn composite electrodes to be competent to show good electrochemical performance, including a stable and long cycling life, low electrochemical impedance, and enhanced specific capacity, which demonstrated a fascinating potential as a promising anode for the next-generation LIBs.

## 2. Experimental Section

### 2.1. Preparation of SiO_2_@Pc Composites Material

Diatomite (325 mesh, Sinopharm Chemical Reagent Co., Ltd. Shanghai, China) was ground for 10 h by a high-energy ball mill, then the sample was dispersed in the glucose aqueous solution by ultrasonic for 15 mi. After that, the freeze-drying process for 60 h was carried out, in which the mass ratio of SiO_2_ to glucose was 1:1 (*w*/*w*). Then, the freeze-drying samples were transferred to a tube furnace and carbonized for 3 h at 500 °C in an Ar/H_2_ gas environment to obtain SiO_2_@Pc composites.

### 2.2. Preparation of SiO_2_@Pc @Sn Composites Material

The previously obtained SiO_2_@Pc from the above step was weighed at ratio of 1:1 (*w*/*w*) with Sn powders (325 mesh, Sinopharm Chemical ReagentCo., Ltd. Shanghai, China) and mixed fully. Then the mixture was transferred to a tubular furnace (OTF-1200X), and heated at a rate of 5 °C/min to 300 °C, keeping for 1 h in an Ar/H_2_ protect gas. After that, the sample of SiO_2_@Pc@Sn was obtained via rapid cooling.

### 2.3. Battery Assembly and Electrochemical Measurements

The Celgard 2320 (Shenzhen, China) film was used as a membrane and lithium foil as a pair electrode to conduct electrochemical experiments on the CR2032 (Shenzhen, China) coin battery. The experimental electrolyte was configured of 1.0 M LiPF_6_ dissolved in ethylene carbonate (EC) and diethyl carbonate (DEC) by volume 1:1. The working electrode was composed of 70 wt.% active materials, 15 wt.% polyvinylidene fluoride (PVDF) binder, and 15 wt.% Super P. After fully mixing and grinding, the slurry was spread on the copper foil evenly, and then dried in an oven at 50 °C for 12 h. The battery was assembled in a glove box filled with Ar gas and the oxygen and water content below 1.0 PPM. After assemblage, the batteries were set aside for 8 h at room temperature. The electrochemical performance was tested by electrochemical impedance spectroscopy (EIS) and cyclic voltammetry (CV) on a DH7001 electrochemical workstation, and the scanning rate of CV was set in the range of 0.1–0.5 mV s^−1^ with an applied potential 2–0 V, and the frequency range for EIS measurement was set in 1.0 MHz–0.1 Hz. All batteries’ simulation cycling and charge/discharge were conducted on a land battery cabinet (LAND CT2001A, Wuhan, China). In the batteries’ evaluation process, the cut-off voltage was 0.005 V vs Li/Li^+^ for discharge and 1.5 V for charge. All specific capacity was calculated based on the proportion of the active material in the whole electrode.

### 2.4. Characterization

The morphology and structure of SiO_2_@Pc@Sn were obtained by a field-emission scanning electron microscope (FESEM, JEOL JMS-7001-F, JEOL, Tokyo, Japan). The element mapping was measured by the EDS instrument equipped in the FESEM. The phase composition of the material was obtained by X’Pert PRO diffractometer (XRD, Shimadzu, Japan: XRD-6000, Cu–K radiation, 0.15406 nm, λ = 1.5406 Å), the measurement angle was between 10–80°, and the scanning rate was 10°/min. Raman spectroscopy was used to characterize the form of carbon, and the excited wavelength of the laser was 532 nm (Raman, InVia and Ntegra Spectra, Renishaw & NT-MDT, London, UK). The thermogravimetry (TG) analysis was performed by the vertical zero friction dilatometer L75VS Linseis (Selb, Germany) from 25 to 800 °C in air to calculate the carbon weight percent in the composite.

## 3. Results and Discussion

**Figure 1:**
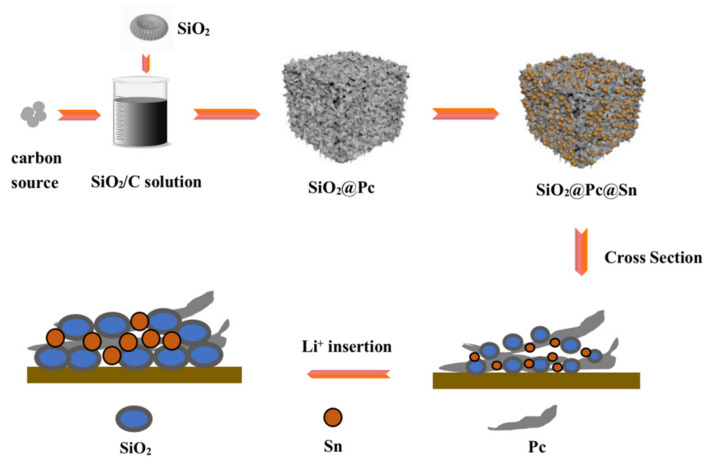
Schematic diagram of the synthesize process of silicon oxide@Pourous Carbon@Tin (SiO_2_@Pc@Sn) composite.

The preparation flow chart of the SiO_2_@Pc@Sn composite is shown in Figure 1. As depicted in the schematic diagram, firstly, the SiO_2_@Pc composite with a porous structure was prepared by the freeze-drying method, and secondly, the SiO_2_@Pc@Sn composite was obtained via the low-temperature thermal melting and self-assembly process.

**Figure 2:**
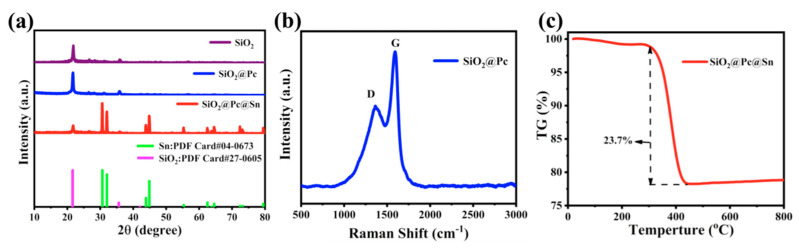
(**a**) XRD patterns of different samples; (**b**) Raman spectrum of SiO_2_@Pc; (**c**) thermogravimetry (TG) diagram for the SiO_2_@Pc.

Figure 2a shows the comparison of the XRD pattern of SiO_2_, SiO_2_@Pc, SiO_2_@Pc@Sn, and PDF card of standard XRD, correspondingly. The characteristic peaks at 21.6° and 35.6° belonged to SiO_2_ [26], and the peak value of SiO_2_@Pc was consistent with that of SiO_2_, indicating that SiO_2_ did not change significantly after Pc coating. In the SiO_2_@Pc@Sn composite, the characteristic peaks for Sn were centered at 30.6°, 32.1°, 43.9°, 44.9°, and 55.3°. The characteristic peaks that belonged to SiO_2_ and Sn in the SiO_2_@Pc@Sn composite were matched well with the standard PDF cards. The synthesized Pc was characterized by the Raman spectrum as indicated in Figure 2. It can be seen that the peaks around 1357 and 1591 cm^−1^ corresponded to the disordered D peak and graphitized G peak for the obtained Pc. The D peak was generally the crystallization defect of carbon atoms and the G peak represented the in-plane vibration of sp^2^ hybridization of carbon atoms [48,49]. The existence of the G peak and D peak indicates that the microstructure of Pc in the SiO_2_@Pc@Sn composite was graphitized carbon. In addition, the main peak of SiO_2_ at 480–490 cm^−1^ was not present in the current Raman spectrum 500–3000 cm^−1^ [27], while the peak around 1080 cm^−1^ under D peak of carbon was also invisible due to the encapsulation of SiO_2_ in a carbon shell [26]. From the TG analysis result in the Raman spectrum, the C weight percent in the SiO_2_@Pc composite was ~27.3%.

**Figure 3:**
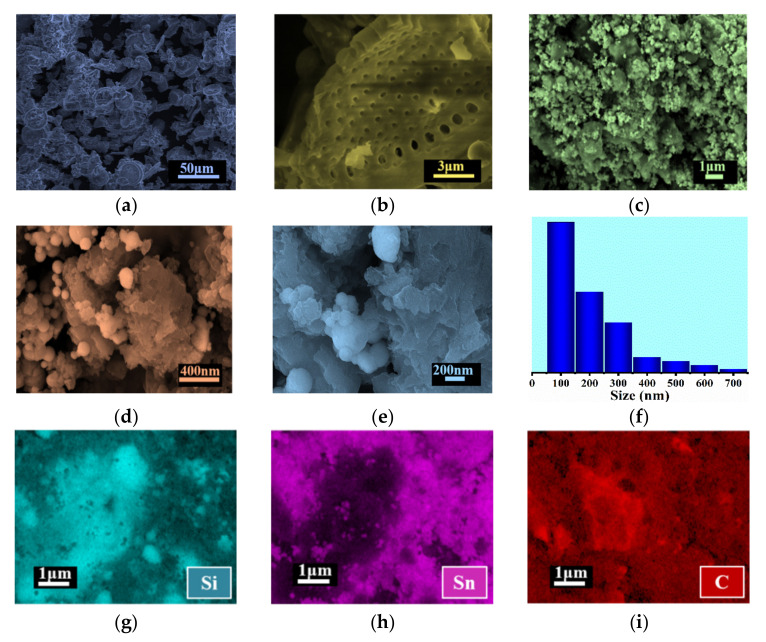
(**a**,**b**) SEM of bare SiO_2_ with different magnification; (**c**–**e**) SEM of SiO_2_@Pc@Sn with different magnification; (**f**) size distribution for Sn in the SiO_2_@Pc@Sn composite; (**g**,**i**) element mapping of SiO_2_@Pc@Sn for Si (**g**); Sn (**h**); and C (**i**).

The morphology and elements distribution of the obtained SiO_2_@Pc@Sn composite was measured by scanning electron microscopy (SEM). As shown in Figure 3, the pristine SiO_2_ was in the shape of a sunflower (Figure 3a), and its average size was between 20–40 μm, with many nano-pores on the surface (Figure 3b). The size of the pores was in the range of 50–600 nm (Figure 3b). From the images as shown in Figure 3c, after rapid cooling, the Sn nano-balls were formed and dispersed uniformly in the SiO_2_@Pc composite, which filled into the pores in Pc or embedded among the SiO_2_@Pc blocks. As shown in Figure 3f, the statistical distribution of size for the Sn balls was mainly centered around 100 nm. The element mapping for Si, Sn, and C in the SiO_2_@Pc@Sn composite is shown in Figure 3g–i. From the result, it is found that three elements are distributed in all the areas detected in the SiO_2_@Pc@Sn.

**Figure 4:**
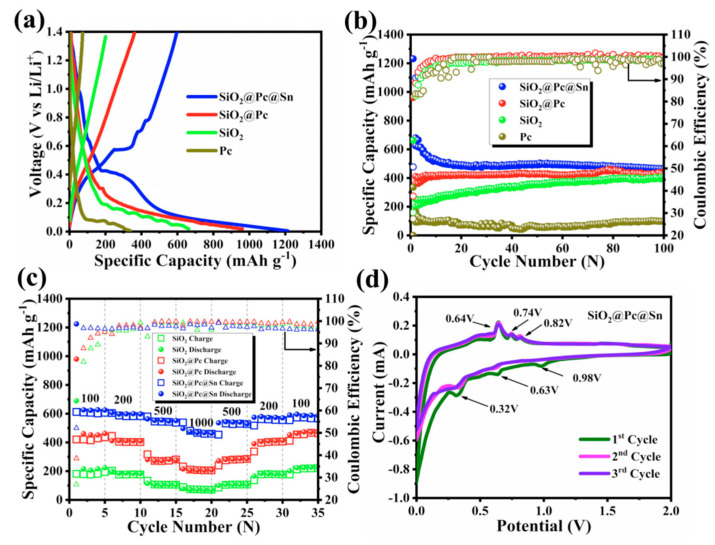
(**a**) Charge/discharge curve of different samples; (**b**) cycling performance curve of different samples; (**c**) the rate performance for different samples; (**d**) cyclic voltammetry (CV) curve of SiO_2_@Pc@Sn.

The electrochemical performance is displayed in Figure 4. The charge/discharge curves of different samples at the same current density of 100 mA·g^−1^ are compared in Figure 4a. It was found that the first discharge capacity reached to 1228 for SiO_2_@Pc@Sn, 990 for SiO_2_@Pc, 672 for bare SiO_2_, and 352 mA·h·g^−1^ for the synthesized Pc. The initial coulomb efficiency (ICE) was 52%, 37.7%, 29.9%, and 27.4%, respectively. The improved specific capacity and ICE of SiO_2_@Pc@Sn were attributed to the fact that Pc and Sn can improve the electrical conductivity of the composite and enhance the electrochemical activity of SiO_2._ The poor conductivity of SiO_2_ was the cause of the low initial coulombic efficiency, and most of Li ions combined with SiO_2_ to produce irreversible Li_4_SiO_4_ and Li_2_O at the first charge and discharge [50,51], while the presence of Pc and Sn improved the whole electrical conductivity of SiO_2_@Pc@Sn, which is helpful for the electrons to arrive at the surface of SiO_2_, and as a result, facilitated the Li ions transfer in the composite. Meanwhile, the existence of Pc further prevented the by-products brought by the direct reaction between electrolyte and SiO_2_ and Sn, thus improving the ICE of the composite [52]. The cycling performance at 100 mA·g^−1^ is compared in Figure 4b. It is evident that SiO_2_@Pc@Sn shows the highest specific capacity and best capacity retention through 100 cycles. While for bare SiO_2_, the capacity underwent continuous increasing during the initial 100 cycles that changed from the initial 200 to 400 mA·h·g^−1^ after 100 cycles. Though the capacity of SiO_2_@Pc could not reach SiO_2_@Pc@Sn, it was still better than bare SiO_2_ and Pc. Moreover, the rate capability for different samples was listed in Figure 4c. It is clear that the SiO_2_@Pc@Sn exhibited capacities of 650, 610, 580, and 520 mA·h·g^−1^ at 100, 200, 500, and 1000 mA·g^−1^, respectively, whereas the bare SiO_2_ and SiO_2_@Pc exhibited a lower capacity and faster capacity decay. Obviously, the rate capability of SiO_2_@Pc@Sn was better than that of the others, especially at high current density due to the fact that Pc and Sn had better conductivity than SiO_2_, which provided higher mobility for Li ion diffusion through the whole electrode. Without the addition of other assistance, such as an electrolyte additive (for instance, FEC) and so on, the good rate capability and stable cycling performance of SiO_2_@Pc@Sn was believed to be originated from the unique structure. Firstly, the built-in void in Pc and SiO_2_ shorted the Li ions transfer distance in the electrode; secondly, Sn and Pc were conductive for electrons and ions, and the face-to-face contact between Pc and SiO_2_ as well as Sn aroused more efficient channels for fast transfer of electrons and Li ions [15,17]. The CV test could detect electrode surface reaction process, electrochemical activity, and reversibility of the active material. Figure 4d is the CV curve of SiO_2_@Pc@Sn. As shown, the cathode peak of 0.98 V in the first cathode scan was caused by electrolyte decomposition and the generation of the SEI layer [50], while the reductive peaks at 0.63 and 0.32 V were attributed to the phase Li*_x_*SiO_y_ and Li*_x_*Si formed while SiO_2_ was combined with Li^+^ in the discharge process [30,40]. On the contrary, the oxidation peaks at 0.64, 0.74, and 0.82 V were caused by the dealloying process of Li*_x_*Sn and Li_2_Si_2_O_5_ [53].

**Figure 5:**
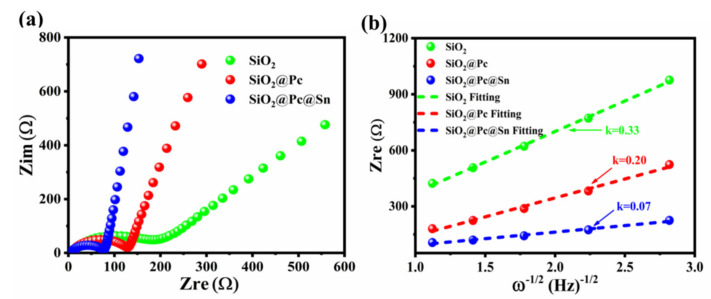
(**a**) Comparison for the Nyquist diagram of different samples; (**b**) impedance real part *Z_re_* Vs *w*^−1/2^ in the frequency range 1–0.1 Hz.

Furthermore, the electrochemical impedance spectra (EIS) were compared and analyzed in Figure 5. From the Nyquist plots diagram of different samples, as shown in Figure 5a, it was found that all impedance spectra consisted of a semicircle in the high frequency region and an inclined line in the low frequency region, which corresponded to the Li^+^ migration resistance and interface contact resistance in the active materials, respectively [54,55]. The impedance resistance was 205 for bare SiO_2_, 129 for the SiO_2_@Pc, and 77 Ω for SiO_2_@Pc@Sn, indicating that the migration impedance of Li ions was minimal in the active material of SiO_2_@Pc@Sn. In addition, EIS was often used in the qualitative determination of Li ions’ diffusion coefficient in LIB materials. Figure 5b is the plots of correlation curve of *Z_re_* (real part of impedance) and *w*^−1/2^ (*w* is the frequency) within the frequency range of 1–0.1 Hz for the electrode composed of different materials. According to the relation, Z_re_ = G−*k·w*^−1/2^, where, *k* is the slope of the correlation curve between *Z_re_* and *w*^−1/2^, from which the diffusion coefficient of lithium ions in different electrode materials can be qualitatively deduced [20]. In order to ensure the accuracy of the experiment, each set of data tested 5–10 batteries for analysis. From the fitting results (Figure 5b), the curve slopes *k* of SiO_2_, SiO_2_@Pc, and SiO_2_@Pc@Sn were 0.33, 0.20 and 0.07, respectively. The result showed that the diffusion coefficient of Li^+^ was the largest in the SiO_2_@Pc @Sn composite according to the relation formula [21], D_Li+_ = A/*k*, where, A is constant related to the Li ions content and electrode area, etc.

**Figure 6:**
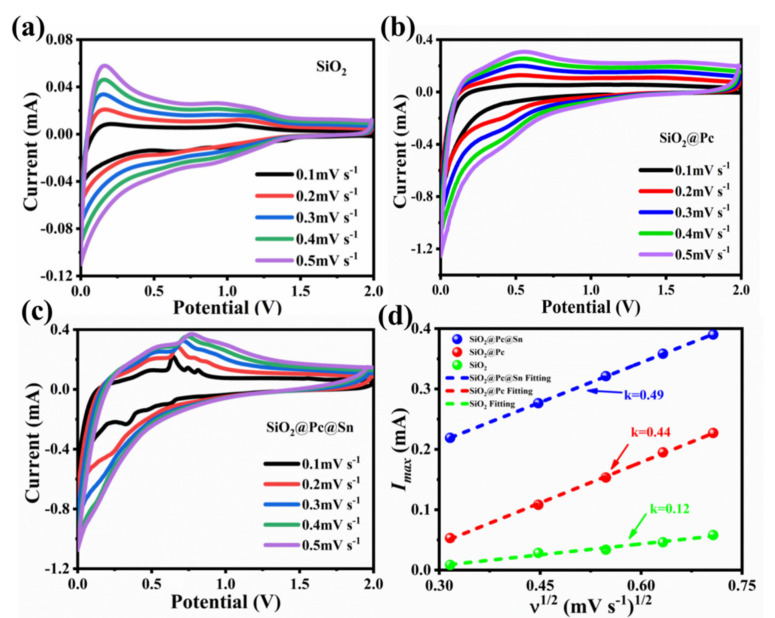
(**a**–**c**) CV curve of SiO_2_, SiO_2_@Pc, and SiO_2_@Pc@Sn with different scanning rates of 0.1–0.5 mV·s^−1^; (**d**) relationship between scan rate and peak current.

Meanwhile, the CV measurement with different scanning rates (mV·s^−1^) is shown in Figure 6. The CV curves of SiO_2_ (Figure 6a), SiO_2_@Pc (Figure 6b), and SiO_2_@Pc@Sn (Figure 6c) electrodes under scanning rates of 0.1, 0.2, 0.3, 0.4, and 0.5 mV·s^−1^ were measured. The obtained peak current *I_max_* and the quadratic root of scanning rate *v*^1/2^ were fitted linearly, from which the diffusion strength of Li^+^ in different electrode materials could be qualitatively determined. According to the Randle–Sevcik equation: *I_max_* = A*v·*^1/2^·D_o*Li*_ [56], where A is the constant related to charge and surface area, *v* is the scanning rate of CV, and D_o*Li*_ is the diffusion coefficient of Li^+^ in oxide [54]. According to the test results shown in Figure 6d, the slopes were 0.49, 0.44, and 0.12 for SiO_2_@Pc@Sn, SiO_2_@Pc, and bare SiO_2,_ respectively, indicating that the diffusion coefficient of Li^+^ ions was the highest in SiO_2_@Pc@Sn compared with the two others [57]. The result was also consistent with the fitting results of the correlation curve between *Z_re_* and *v*^−1/2^ of EIS in the frequency range of 1–0.1 Hz (Figure 5b), which further demonstrated that Sn could improve the electrochemical performance of SiO_2_-based anode materials for LIBs.

## 4. Summary

The SiO_2_@Pc@Sn composite anode material was prepared by the freeze-drying and low-temperature thermal melting method, which exhibited improved electrochemical performance and faster Li^+^ transfer kinetic. The synergetic effect of porous carbon, SiO_2_, and Sn endows the as-fabricated SiO_2_@Pc@Sn composites to be competent to show good electrochemical performance. When used as an anode in LIBs, the SiO_2_@Pc@Sn composite could deliver a large reversible capacity of 650 at 100 mA·g^−1^, a remarkable rate capability of 500 retained at 1000 mA·g^−1^, and a long-term cycling durability with ~87% capacity retention over 100 cycles. EIS and CV measurements demonstrated that, with the participation of Sn phase and Pc, the diffusion and migration kinetics of Li ions in SiO_2_@Pc@Sn composites was significantly improved. The understanding of the synergistic effect of Li storage between SiO_2_ and Sn in this work will not only provide insight towards exploring new SiO_2_-based anode materials, but also shed light on the design of other low-cost and environmentally friendly electrode materials for the next-generation LIBs.

## Data Availability

Data sharing not applicable.
